# The impact of an encounter with a gynaecologic dermatologist on quality of life, health literacy and education satisfaction for patients with vulvar lichen sclerosus: A survey study

**DOI:** 10.1002/ski2.89

**Published:** 2021-12-30

**Authors:** E. M. Kolitz, J. Pineider, M. M. Mauskar, A. Rutherford

**Affiliations:** ^1^ University of Texas Southwestern Medical Center University of Texas Southwestern Medical School Dallas Texas USA; ^2^ Department of Dermatology and Obstetrics & Gynecology University of Texas Southwestern Medical Center Dallas Texas USA; ^3^ Department of Dermatology University of Texas Southwestern Medical Center Dallas Texas USA

## Abstract

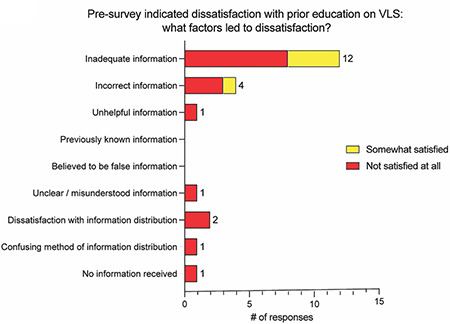

1

Dear Editor,

Vulvar lichen sclerosus (VLS) is a chronic inflammatory dermatosis which can severely impact quality of life (QOL).^1^, ^2^, ^3^, ^4^ Women with VLS express disappointment in medical care and view this as a social justice issue.^2^, ^3^ Reasons for unsatisfactory care are multifactorial, with societal, health system and condition‐specific factors implicated.^2^, ^3^, ^5^ Ultimately, patients are eager to learn about their VLS and associate quality care with communication, treatment and education.^2^, ^3^ However, decreased health literacy negatively impacts a patient's healthcare experience and may diminish compliance.^5^ The purpose of this study is to investigate QOL, health literacy and education satisfaction in VLS patients after an initial encounter with a gynaecologic dermatologist (‘specialist’).

A cross‐sectional pilot survey was emailed to consenting adult women referred to the specialist for VLS. The specialist was blinded to participants. A history and physical were completed including taking patient photographs (Appendix S1). After diagnosis confirmation, the specialist educated the patient. The patient's photograph was printed and annotated for her reference. The specialist demonstrated with petrolatum how much topical medication to use by applying the correct amount on the dorsal hand. A standardized handout, research article and individualized written directions were provided (Appendix S1). Post‐encounter surveys were collected electronically and matched to pre‐encounter surveys using anonymous identifiers. In the survey, QOL was measured using the vulvar quality of life index (VQLI).^1^ Health literacy was assessed with self‐rated disease understanding and management capabilities.^6^ Patients' satisfaction with provider–patient interactions and education was queried. Survey questions were adapted from a health literacy survey study for hidradenitis suppurativa.^6^ A Likert scale (‘not at all satisfied’ to ‘completely satisfied’) assessed education satisfaction in pre‐ and post‐surveys. Ordinal and continuous data were analysed using a non‐parametric Wilcoxon matched‐pairs signed rank test in GraphPad prism version 9.0.0. *p*‐Values are reported for tests of statistical significance. All comparisons were two‐tailed and the alpha error was set at 5%.

A total of 31 women with VLS received the survey: 27 (87.1%) completed a pre‐survey and 25 (80.6%) completed a post‐survey. Only matched pre‐ and post‐surveys, with complete responses were included, narrowing the sample size to 15 (48.5%). Two women identified as Hispanic, LatinX or Spanish origin; 13 women identified as White/Caucasian. The average age was 48. After the encounter, VQLI decreased significantly, indicating improvement in QOL (*p* = 0.0083). Patient‐reported health literacy significantly improved after their encounter as determined by patient‐rated knowledge (*p* = 0.0004), ability to manage (*p* = 0.0002), tools to treat (*p* = 0.0005), ability to explain VLS (*p* = 0.0039) and amount of education provided (*p* = 0.0012). Survey responses prior to the patient encounter indicated patients were less than satisfied with previous education by healthcare providers with 67% of patients reported to be ‘not satisfied at all’ and 27% ‘somewhat satisfied’ (Figure 1a). After the encounter, data showed a statistically significant improvement in patients' satisfaction rating regarding explanation of disease complications, treatment goals and treatment duration, with 53% of individuals reported to be ‘completely satisfied’ and 33% ‘very satisfied’ (Figure 1b). Results indicated education was helpful, trustworthy and delivered suitably. Overall, all participants acknowledged a positive experience with the specialist compared to previous medical encounters. Patient's indicated that using the patient's own photograph for education (93%), demonstrating where to apply medication (93%), demonstrating amount to use (87%) and listening to patient experiences (87%) were the differentiating factors for the satisfactory specialist encounter (Figure 1c,d).

**FIGURE 1:**
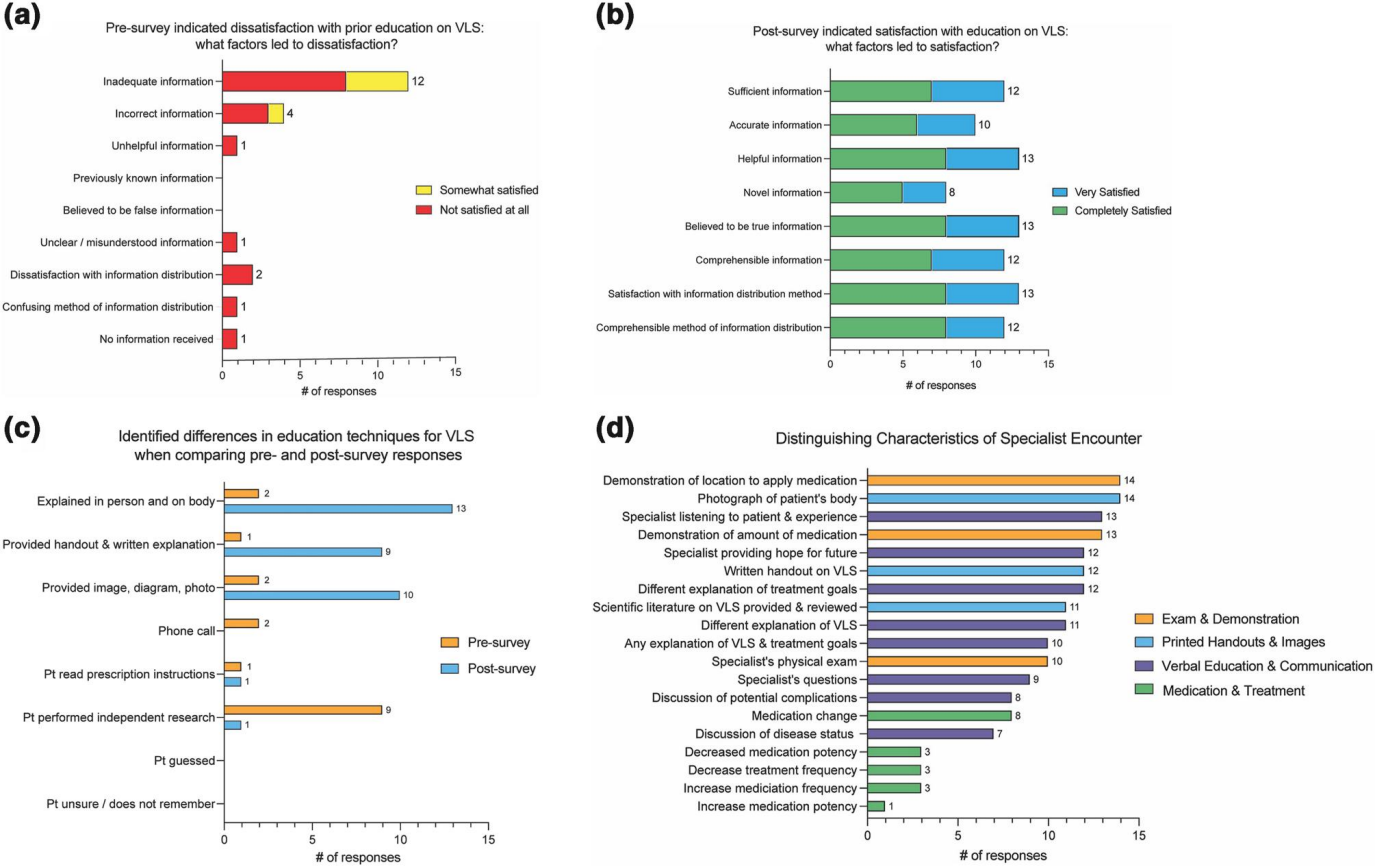
Distinguishing factors impacting patient education and satisfaction reported by patients with vulvar lichen sclerosus at initial gynaecologic dermatology specialist encounter. (a) Factors that led to dissatisfaction with education for patients who answered ‘not satisfied at all’ (red, *n* = 10) or ‘somewhat satisfied’ (yellow, *n* = 4). One patient did not respond. (b) Factors that led to satisfaction with education if patients answered ‘completely satisfied’ (green, *n* = 8) or ‘very satisfied’ (blue, *n* = 5). Two patients responded either ‘somewhat satisfied’ or ‘satisfied’, not included on table. (c) Different methodologies of how information was presented or given to the patient at previous medical encounters (orange) versus the specialist (blue). (d) Factors that >80% (*n* ≥ 12 of 15) of participants selected differentiating the specialist encounter from previous medical encounters

This study confirms prior findings that demonstrate VLS specialist encounters lead to improved QOL.^4^ Furthermore, this study revealed a specialist encounter improved patients' health literacy and education satisfaction. Previous reports show patients with VLS may not receive adequate information or education about their condition before speciality referral.^2^ By addressing patients' concerns regarding their prior experience in health care,^2^ we were able to differentiate characteristics of the visit that increased understanding of VLS, confidence in management and overall satisfaction. Characteristics, such as patient demonstration of topical treatments and utilizing the patient's own photograph for personalized education, left significant impressions on patients. Since treatment non‐adherence in this population relates to misunderstanding of treatment quantity and location,^2^, ^7^ the simple techniques described above may potentially address adherence challenges.^5^, ^8^ It is our hope that these findings can be implemented amongst all healthcare providers that encounter these patients.

Limitations of this pilot study include small sample size and selection, recall, and observation biases. Annotated patient photographs and demonstrations of how much and where to apply topical treatments can be incorporated into encounters for other dermatologic conditions requiring topical treatments. Future studies should assess whether these techniques translate to improved patient treatment adherence and clinical outcomes in VLS.

## CONFLICT OF INTEREST

The authors declare no conflict of interests.

## AUTHOR CONTRIBUTIONS

**E. M. Kolitz:** Data curation; Formal analysis; Resources; Writing – original draft; Writing – review & editing. **J. Pineider:** Data curation; Project administration, Resources, Writing – original draft; Writing – review & editing. **M. M. Mauskar:** Conceptualization; Supporting; Investigation; Methodology; Project administration; Resources; Supervision; Writing – original draft; Writing – review & editing. **A. Rutherford:** Conceptualization; Formal analysis; Investigation; Methodology; Project administration; Resources; Supervision; Writing – original draft; Writing – review & editing.

## Supporting information

Supporting Information S1

Supporting Information S2

## Data Availability

Data sharing not applicable to this article as no datasets were generated or analysed during the current study.
