# Supplementary material for: The impact of an encounter with a gynaecologic dermatologist on quality of life, health literacy and education satisfaction for patients with vulvar lichen sclerosus: A survey study
Source: Skin Health Dis. 2021 Dec 30;2(3):e89. doi: 10.1002/ski2.89 (PMC9435454; doi:10.1002/ski2.89)
Supplement: Supplementary file 1 — Supporting Information S1 [file SKI2-2-e89-s002.docx]

Lichen Sclerosus (LS)

Key Points

- Lichen sclerosus is a chronic disease, but it can be managed with topical corticosteroids.
- The initial goal of therapy is to calm active disease with high-potency steroids. Even once active disease is in remission, you will continue to use steroids (less potent and less frequently) for maintenance therapy to prevent recurrence, scarring, or vulvar cancer.
- Topical steroids are the only therapy proven to treat active disease and prevent progression to cancer, and they work very well for this condition.

Lichen sclerosus (LS; sometimes called lichen sclerosus at atrophicus) is a skin condition that is most common on the vulva of older women who have gone through menopause. However, LS also affects girls before puberty as well as young adult women. Approximately 20% of the women diagnosed with LS are between the ages of 18 and 45. LS often affects rectal skin also. Only about one in 30 women with vulvar LS experience LS on skin away from the genital area; this is usually on the back, chest, or abdomen, and almost never on the face or hands.

The causes of LS are not completely understood. Autoimmune and genetic factors likely play a role. The immune system, the part of the body which fights off infection, becomes over-active and attacks the skin by mistake. Why this happens is not known, but it is not because of anything you did or didn’t do. Because this is an autoimmune condition, it frequently occurs in patients with other autoimmune conditions, such as vitiligo or hypothyroidism. It may be appropriate to screen for other autoimmune conditions in some patients. Lichen sclerosus runs in families in about 10% of cases, so genetics may also be a factor.

Lichen sclerosus of the vulva or perianal region can cause the top layer of skin to appear thin and white, and it is very itchy. The skin is also fragile, so that rubbing and scratching can cause breaks, cracks, and bruises which then hurt. Sexual activity is often painful. Untreated LS eventually can cause scarring of the labia minora and clitoris and narrowing of the opening of the vagina. There is also a small risk of skin cancer of the vulva, which occurs in less than 5% of patients. Well-controlled LS is at much less risk for both scarring and cancer.

Lichen sclerosus usually improves very quickly with appropriate treatment. The best treatment is a strong topical corticosteroid ointment, such as clobetasol or betamethasone. The corticosteroid is applied very sparingly once or twice a day to start, depending on how active your disease is. The amount of medication used should be about the size of a lentil (smaller than a pea). If the skin feels greasy after medication is applied, too much is being used. When too much medication is used, or when medication is used for too long without careful follow-up, the skin can thin and become irritated and red. However, corticosteroids are very safe medications when used in the correct amounts and for the correct length of time. Therefore, a healthcare provider should examine the area regularly while medication is being used daily to assess for progress and side effects. The itching and irritation usually improve within the first week. With ongoing use of the corticosteroid, the color and strength of the skin gradually return to normal.

LS usually is well controlled with a corticosteroid, but it is not cured. Therefore, if the medication is stopped, itching and irritation reappear. Scarring continues, sometimes even before itching or irritation return. Daily use of the corticosteroid usually is needed for two to four months for the normal color and strength to return. When the skin is controlled, your healthcare provider will talk to you about reducing the frequency of the steroid application down to three times per week, and/or switching to a less potent steroid (such as triamcinolone or desonide). In the past, patients were treated as needed for disease. However, studies have shown that maintenance therapy tailored to individual patients’ disease activity is required to prevent skin cancer.

It is also important to consider other factors which may cause vulvovaginal irritation, such as any vaginal infection, low estrogen, or substances that irritate the area. These include creams, unnecessary medications, soaps, and over-washing. Washing should be limited to once a day with clear water only. Some irritants, such as sweat or urine, can be hard to avoid, but efforts should be made to keep the area cool and dry. Referral to urology or pelvic floor physical therapy can help with incontinence. A zinc oxide cream (such as Desitin or Boudreaux’s butt paste) can help prevent irritation from moisture.

Rubbing and scratching also irritates the vulva. Many people can keep from scratching during the day, but rub and scratch at night without realizing it. A medication that produces a very deep sleep can stop nighttime scratching and allow the skin to heal. Additionally, anti-depressant medications at very low doses can help with irritation and nerve pain. Sometimes, women with LS experience a pain syndrome called vulvodynia triggered by the irritation of the nerves from their disease. If this happens, several options exist, including pelvic floor physical therapy, mindfulness exercises, and some medications.

Several other therapies have been studied for lichen sclerosus, but none are superior to topical corticosteroids. Topical tacrolimus (Protopic^R^) or pimecrolimus (Elidel^R^) may be an option for patients who do not respond to steroids, but they often burn when applied and steroids are more effective in clinical trials. You can explore this option with your healthcare provider if topical steroids do not seem to be working, but most often they work quite well. Other investigative treatments include lasers (e.g. fractioned CO^2^ laser/MonaLisa Touch^R^) or platelet-rich plasma (PRP), but trials have not shown these therapies to be proven effective in treating LS symptoms or preventing progression to vulvar cancer. These procedures can be costly and painful, and are currently considered experimental. Future research is needed to determine whether these are appropriate for treatment of LS. If you have questions about these therapies, please discuss them with your provider.

Nearly all patients do extremely well following treatment for LS. Even when LS is completely controlled, however, patients should be followed every six months to be sure the disease remains controlled, to detect side effects of the medication, and to examine for very early skin cancers.
