# Supplementary material for: The impact of an encounter with a gynaecologic dermatologist on quality of life, health literacy and education satisfaction for patients with vulvar lichen sclerosus: A survey study
Source: Skin Health Dis. 2021 Dec 30;2(3):e89. doi: 10.1002/ski2.89 (PMC9435454; doi:10.1002/ski2.89)
Supplement: Supplementary file 2 — Supporting Information S2 [file SKI2-2-e89-s001.docx]

Standardized New Patient Visit

Vulvar Lichen Sclerosus

- Introduction of provider
- Conversation and History:
  - Review patient’s responses on handout and ask additional questions (make note of what they do for work, family, etc.)
  - Ask about hygiene, washing, product use, wet wipes, etc.
  - If they are on topical therapy already ask how they use this and what they were told about steroid
  - If they have seen multiple providers or been symptomatic for extensive time, discuss and ask what they understand about the condition
    - This is usually where they tell me about chat rooms or online groups if they are part of one, I also know this based on some of the topicals they are doing
  - Depending on the patient, set the ground work, especially if they have seen many health care professionals. For example, “by the time someone gets to this clinic, they usually have more than one thing going on. If it was just ‘one thing’ they probably would not be here, or they would have gotten better.”
- Exam:
  - Position patient and feet in foot rests
  - Evaluate vulva systematically
  - Take photo with patient consent and explanation of purpose
  - Obtain Wet prep and KOH
  - Have patient point to pain / or itching spot
  - Have patient show with Vaseline how much and where they are applying it topical steroids or topical medications
  - Decide if patient needs biopsy.
  - Let patient relax, sit up out of the foot rests
- Step out of room, collect thoughts, review wet prep and KOH under microscope
- Education and Treatment Plan:
  - Return to exam room, discuss with patient wet prep/KOH, what was discovered on physical exam, considerations for diagnoses or contributing factors, and next steps
    - IF biopsy needed: Will apply topical lidocaine and discuss biopsy expectations.
  - Review what they may be doing already that could be modified (aggressive cleaning habits, topical, wet wipes, incontinence)
  - If clinically consistent with LS or suspect highly LS: Will have assistant bring handout on LS (generic patient info) and article on importance of compliance (Lee 2015 JAMA derm)
  - Give printed photograph:
    - Talk about any findings they have referencing photo
    - Annotate with how much and were they should put medication with skin pen / marker
    - Write on the side of the photo application instructions as well as draw a small circle demonstrating the size of the “1/2 pea or lentil” vs other amount they should use
  - Demonstration: Take Vaseline, show them how much to apply on the tip of my finger
    - Then rub this amount on the back of the hand so they can see how far a small amount will go. Have them feel the back of the hand to see what they should feel like after they apply medication
  - Discuss that in an ideal world, the first 1-2 times they apply the medication, they should put their photo against the wall and next to a mirror to see where to apply the medication compared to their vulva. Tell them that people get into trouble with steroids when they use too much in the wrong place. If they do what is discussed, the only “side effect” will be that they will get better!
  - Review research article (Lee), summarize findings. Show them photos of other patients in the article with LS and where they compare to this.
  - Tell them this is pre-cancerous and an evaluation should be completed every 6 months to monitor for disease activity and skin cancer
  - Give patients opportunity for questions / comments
- Closed circle communication:
  - Step out of room, have patient get dressed if they have not already done so, have them read generic patient handout
  - Ask them to write any questions they to review together before they leave
  - Type up instructions on the After Visit Summary and send in medication prescriptions
  - Go back in room, answer any final questions, go over instructions one last time
- Ask them to sign up for MyChart (or alternative electronic patient portal) so they can send questions if needed or discuss how to call clinic if they prefer
- Have patient return in 4-12 weeks depending on activity / severity / etc.
